# Microsaccades Distinguish Looking From Seeing

**DOI:** 10.16910/jemr.12.6.2

**Published:** 2019-06-01

**Authors:** Eva Krueger*, Andrea Schneider*, Ben D. Sawyer, Alain Chavaillaz, Andreas Sonderegger, Rudolf Groner, P.A. Hancock

**Affiliations:** University of Central Florida, USA; University of Bern, Switzerland; Massachusetts Institute of Technology, USA; University of Fribourg, Switzerland; École Polytechnique Fédérale de Lausanne (EPFL), Switzerland; *These authors contributed equally to the work.

**Keywords:** Fixational eye movements, eye tracking, microsaccades, visual load, visual attention

## Abstract

Understanding our visual world requires both looking and seeing. Dissociation of these processes can result in the phenomenon of inattentional blindness or ‘looking without seeing‘. Concomitant errors in applied settings can be serious, and even deadly. Current visual data analysis cannot differentiate between just ‘looking‘ and actual processing of visual information, i.e., ‘seeing‘. Differentiation may be possible through the examination of microsaccades; the involuntary, smallmagnitude saccadic eye movements that occur during processed visual fixation. Recent work has suggested that microsaccades are post-attentional biosignals, potentially modulated by task. Specifically, microsaccade rates decrease with increased mental task demand, and increase with growing visual task difficulty. Such findings imply that there are fundamental differences in microsaccadic activity between visual and nonvisual tasks. To evaluate this proposition, we used a high-speed eye tracker to record participants in looking for differences between two images or, doing mental arithmetic, or both tasks in combination. Results showed that microsaccade rate was significantly increased in conditions that require high visual attention, and decreased in conditions that require less visual attention. The results support microsaccadic rate reflecting visual attention, and level of visual information processing. A measure that reflects to what extent and how an operator is processing visual information represents a critical step for the application of sophisticated visual assessment to real world tasks.

## Introduction

When humans attend to their surrounding environment, looking does not always equate to seeing. That is, the externalities of the visual process do not always correspond to the attended percept. Historically, visual attention has been measured predominantly using eye fixations (1, 2). The implicit assumption here is that fixating an object secures visual attention and allocates mental resources. However, fixations do not necessarily imply attentional focus (2, 3, 4).

Looking without seeing can give an explanation for various phenomena of inattentional blindness, which have been reported beyond the laboratory in a number of applied domains such as surface transportation (5), baggage screening (6), and surveying crowds (7). As an example of these real-world scenarios, consider a driver who is stopped on the roadway, their eyes directed toward a red signal. The signal turns green, but the driver fails to react. As they wait, eyes directed toward a signal that is now green, we can understand that they are certainly passively ‘looking‘ at the light. Further, if they fail to respond, they cannot be said to have processed the change from red to green and thus to have ‘seen‘ the signal. Looking without seeing is a phenomenon which should be explained by workable theories of human information processing, most notably models of attention. However, apart from a behavioural reaction, no measure allowing for an objective distinction between looking and seeing has been suggested so far. The present work evaluates the utility of microsaccades as an indicator of visual attention and its underlying sensory and physiological processes in order to distinguish between looking from seeing by using a replicable and quantitative measure. In the present context, “paying attention” is considered a top-down regulated mechanism of allocating processing resources to parts or properties of the input on cost of other (see the taxonomy of attentional processes in Groner & Groner, 2000). Microsaccades will be investigated as possible indicators of such a process of resources allocation.

Microsaccades represent small, involuntary eye movements, similar to miniature versions of voluntary saccades. Typically, microsaccades have an amplitude less than two degrees of visual angle (8, 9). Microsaccades occur during visual fixation in the period of relative stability between the larger saccades. Even when we think that our eyes are not moving, they are. Microsaccades are not under voluntary control, and therefore they are more robust with respect to external influences (9, 10). The functions of microsaccades are not yet fully understood. Research has focused on the relation between microsaccades and the control of fixation position, reduction of perceptual fading, continuity of perception, visual acuity, scanning of small spatial regions, shifts of spatial attention and resolving perceptual ambiguities (8, 11). Recent results challenge the interpretation of microsaccades as strictly low-level oculomotor phenomena (11). Accumulating empirical evidence is beginning to confirm that microsaccades serve both perceptual and oculomotor goals. A direct link between microsaccade production and visibility has been shown; increased microsaccade production during fixation results in enhanced visibility for peripheral and parafoveal visual targets (12). Decreased microsaccade production leads to periods of visual fading (13). Several studies have found that microsaccades, like saccades themselves, can be modulated by attention. For instance, the spatial location indicated by an attentional/visual cue can bias microsaccade directionality (10, 14). This is most likely due to the extensive overlap between the neural systems that control attention and the system that generates saccadic eye movements. Martinez-Conde et al. (2009) have suggested production or control of microsaccadic activity by attentional processes, toward the goal of improving vision through dynamic enhancement and suppression of low-level visual information over time. Such suppositions require further investigation, but these existing results suggest that microsaccadic activity could be a robust biosignature for internal attentional processes.

Microsaccades activities are influenced by the attentional load of visual tasks (15, 16) as well as non-visual cognitive tasks (17, 18, 19). These, non-visual cognitive tasks include arithmetic operation and digit retention, and are intended to involve mental processes that do not rely on vision. However, the growing body of literature on attentional load and microsaccade rate is inconsistent. Some studies indicate that tasks with higher attentional load lead to a lower microsaccade rate. For example, (20) found higher attentional load associated with lower microsaccades rates and increased microsaccade directional congruency. Their paradigm employed visual recognition tasks requiring either low attentional load (reporting color) or high attentional load (reporting letter shape). 17 found increasing task difficulty to correspond to lower microsaccade rate, using a paradigm which employed a mental arithmetic task, lacking any visual component. 18 performed a subsequent replication, which also showed an inverse relationship between the microsaccade rate and task difficulty. 19 used two-digit (low load) and five-digit (high load) number memorizing tasks to investigate the association between the working memory load and the microsaccade rate. In line with these previous studies, they revealed that the microsaccade rate was significantly suppressed in the task with high working memory load. However, still other studies have found microsaccade rate increases with increasing task demand. 15 employed a simulated driving task using a low load task (control task) and a high load task (dual task including visual search task). They found significantly more microsaccades under the high load condition. 16 used a forced choice-task paradigm. Participants had to judge the orientation of a titled stimulus that was placed in static or dynamic backgrounds. A higher microsaccade rate was found when participants were engaged in the high load task, in which execution of the discrimination task was needed, compared to the low load task, in which no response was needed.

Under the assumption that complicated interactions between the effects of perceptual and working memory load could occur, 37Xue, Huang, Ju, Chai, Li and Chen (2017) conducted an experiment with monkeys using a task with primarily perceptual load being manipulated. Results indicated that microsaccade rate was lower with high load than with low load. They conclude that the perceptual costs or benefits of microsaccades might drive the observers to adjust their fixation strategies to facilitate behavior performance.

In summary, previous results have shown that a) tasks which induce mostly cognitive load are linked with a decreased microsaccade rate (17, 18, 19) and that b) increasing difficulty in tasks with a strong but not exclusive visual component enhances microsaccade rate (15, 16). This potentially implies that microsaccades are a top-down regulated mechanism of allocating processing resources to parts or properties of input at cost of other processes. In applied settings, this potentially means that microsaccades would indicate whether a person was paying attention to a visual scene or if their attention had shifted to some other cognitive task.

### The present study

To evaluate the assumption that microsaccade rate reflects the amount of visual attention, visual and non-visual attention were manipulated systematically in this study. To investigate this question, a dual task setting with tasks inducing 1) cognitive and 2) visual load was employed. Visual load was defined as the level of complexity of a visual scene relying on the attributes of a visual scene (21). Thus, an environment in which participants would find it difficult to differentiate between important visual cues and irrelevant visual elements was considered “high visual load”. The systematic combination of both tasks allows for an analysis of relations between visual attention and microsaccade rate. We hypothesize that microsaccade rate is increased in trials with high visual load and low mental load. Furthermore, we anticipate that microsaccade rate will decrease in trials with a low visual load and a high mental load.

## Method

### Participants 

Eighteen participants, nine male, nine female, with an average age of 21 years (SD ± 2.56) participated in one single experimental session. All participants were University of Central Florida (UCF) students and received class credit for their participation. All had normal or corrected-to-normal vision, as tested by a Snellen eye chart (22). Experiments were carried out in conformity with the declaration of Helsinki, as well as the appropriately accredited Internal Review Board (IRB) policies. Written informed consent was obtained from each participant prior to the commencement of testing.

### Experimental Design

A 3 x 3 repeated measures design was used in this study. Visual demand (free view vs. easy view vs. hard view) and mental demand (no count vs. easy count vs. hard count) were manipulated as independent variables (see Figure 2), with ‘free view’ and ‘no count’ conditions representing control conditions. The order of the different experimental cells was randomized for each participant.

### Stimuli and Tasks

Visual stimuli representing three different complexity levels were used to manipulate visual load. For the ‘easy view’ and ‘hard view’ conditions, ‘spot the difference’ puzzles were used. While in ‘easy view‘, stimulus material consisted of simple line drawings, photographs with complex visual information were used for the ‘hard view‘ condition.

**Figure 1.**
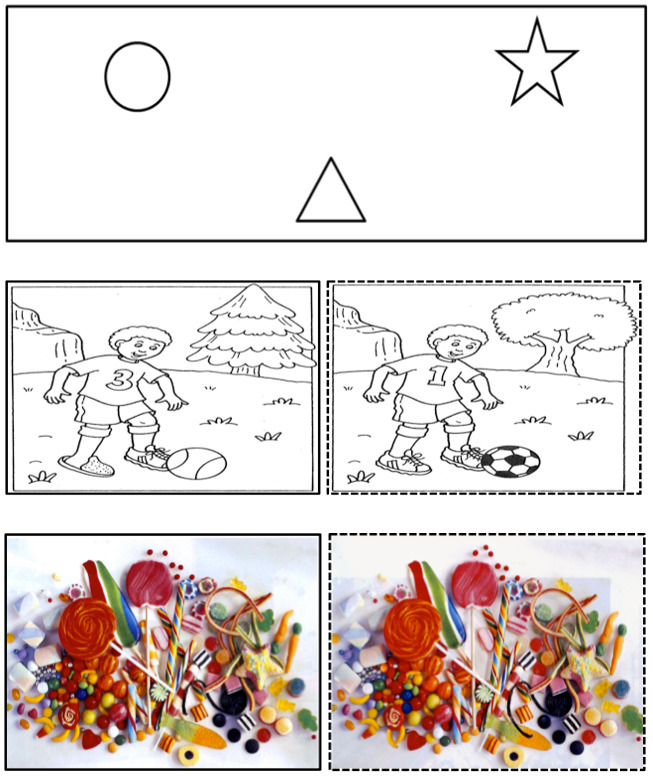
(Upmost) Example of the stimuli - ‘free view‘ condition, (middle) example of the stimuli - ‘easy view‘ condition, (below) example of the stimuli - ‘hard view‘ condition.

The tasks for the ‘hard‘ and ‘easy view‘ conditions consisted of determining differences between the two images displayed next to each other. In ‘easy view‘ condition, such differences were simple to detect, while in the ‘hard view‘ condition, differences were much more difficult to detect (see Figure 1). In the control condition representing the lowest level of visual load (i.e. the free view condition), stimuli consisted of contained three simple geometric forms. This condition involved no visual search task, participants were simply asked to view the images. In order to provide as natural a task as possible, no center target was provided. Ten examples of each type of stimuli were used, one in training and nine in the experiment.

**Figure 2.**
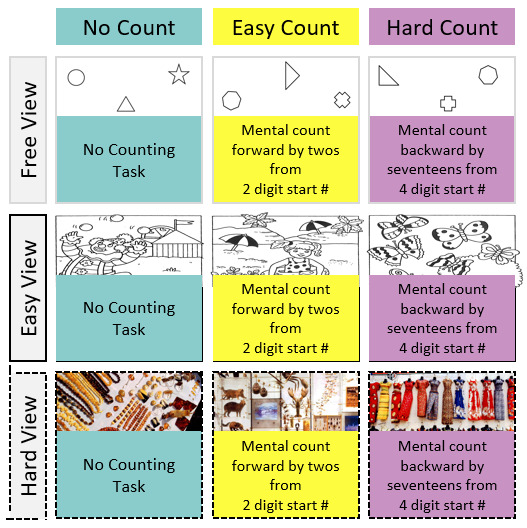
Three levels of difficulty in visual and cognitive tasks resulted in a total of nine conditions presented in the experimental portion of the work.

In order to manipulate cognitive load, participants were asked to complete mental arithmetic tasks while performing the visual search tasks described above. In the ‘easy count‘ condition, participants were instructed to count forward by increments of 2, starting from a random two-digit number.

In the ‘hard count‘ condition, participants counted backward by increments of 17, starting from a random four-digit number (e.g., 3123). In the control condition (i.e. no count), participants were instructed not to count and pay full attention to the picture.

Visual tasks and mental arithmetic tasks were always presented in combination, summing to nine experimental conditions. The ‘no count’ and ‘free view’ conditions represent control conditions in which no formal task was completed. Thus, pairings of conditions including one of these control conditions can be considered as single tasks whereas all the others represent dual tasks. Both tasks have been used in previous studies (17, 23).

### Measures and Instruments

Performance was measured for both the visual task and the arithmetic task. For the visual task, the percentage of total available differences detected in each puzzle was calculated.

In the counting tasks, participants were holding a game controller in both hands. As participants completed each cycle of counting, they pressed a button on the controller. These button presses were recorded by a purpose built program (MCT (Mental Count Timer), Sawyer, 2017). This made it possible to monitor whether participants continually performed the task without requiring them to vocalize, and therefore cause interference with eye tracking. At the end of each trial, participants reported the number at which they had arrived. Answers were scored as either correct or incorrect, based upon the number of iterations reported through MCT combined with the increment required by the counting task (2’s or 17’s).

Eye position was detected binocularly and noninvasively with a video-based eye tracker at 1000 HZ (EyeLink 1000, SR Research, instrument noise 0.01º RMS). In a screening process (for details see 17), erroneous (i.e. temporary intermittent signal) eye position data was first identified and then discarded. In addition, portions of data where very fast decreases and increases in pupil area occurred were extracted (> 50 units/sample, such periods are thought to represent semi-blinks where the pupil is never fully occluded (24). Also, blink periods as portions of the raw data where pupil information was missing were identified and removed. Before and after each blink/semi-blink interval 200 ms were added to eliminate the initial and final parts where the pupil was still partially occluded (24). After the rectifying the eye position data, saccades were identified with a modified version of the algorithm developed by Engbert and Kliegl (9, 14, 25, 26) with λ = 6 (used for the velocity threshold detection) and a minimum saccadic duration of 6 ms. Only binocular saccades (saccades with a minimum overlap of one data sample in both eyes (25, 26, 27, 28)) were considered in order to reduce the amount of potential noise. In addition, a minimum intersaccadic interval of 20 ms was applied with the intention of not categorizing new saccades as potential overshoot corrections (29). Saccades with magnitude < 2º in both eyes were defined as microsaccades (8, 12, 13, 24, 30, 31). Finally, to calculate microsaccade properties such as magnitude and peak velocity, the values for the right and left eyes were averaged.

In order to assess mental workload subjectively as part of a manipulation check, the NASA-Task Load Index (32) was administered after each trial. This subjective multidimensional assessment tool measures perceived workload with six subscales: mental demand, physical demand, temporal demand, performance, effort and frustration on a scale ranging from 1 (very low) to 20 (very high), with performance using verbal anchors ranging from ‘perfect’ to ‘failure’. The scale is widely used in human factors research (33, 34) and has good psychometric properties (c.f. 32).

### Apparatus

The room in which the experiment was conducted was quiet, and equal illumination was used for each session. Participants were placed in a head/chin support, facing a desktop-mounted EyeLink 1000 eye tracker capable of 1000 Hz binocular tracking. Fifty-seven cm away from the support, visual stimuli were displayed on a linearized video monitor (Barco Reference Calibrator V, 75 Hz refresh rate), using SR Research Experiment Builder.

### Procedure

Participants first engaged a training session, which exposed them to each of the experimental manipulations individually and allowed them to ask questions. The experimental session contained 3 blocks, each containing 9 trials, one per experimental condition. For each participant, the trial sequence was randomized. Each trial was 60 seconds in duration, resulting in a total of 27 min of eye-tracking data per participant.

Before each trial, an instruction screen indicated the task which was to be performed. During the free view condition, participants were instructed to look at the picture on the screen, with no search for differences or any specific response being required from them. For the mental arithmetic task, participants were instructed to push a gamepad key with their index finger each time they counted (i.e., either 2 or 17). For the ‘no count‘ task, participants were instructed not to count and pay full attention to the picture. After each trial, participants completed the NASA-Task Load Index. After completion of each block, a five-minute break was administered.

Each visual task had an arithmetic counterpart (see Figure 2). Tasks were always presented in combination, summing to nine total conditions, each a unique combination of visual and arithmetic tasks. The ‘no count‘ and ‘free view‘ condition is essentially the absence of any formed task. Pairings of conditions that include one of these ‘non-tasks’ can be considered as single task.

### Data Analysis

Microsaccade rate and performance data met the assumption of normality (via the Shapiro-Wilks test, all *P*-values > .05), and all data were normally distributed. The dependent variable was microsaccade rate and on this variable we performed a 3 x 3 (no view, easy view, hard view x no count, easy count, hard count) repeated measures MANOVA. Mauchly’s test indicated that the assumption of sphericity is violated (χ²(2) = 29.65, *p *< .001), therefore degrees of freedom were corrected using Greenhouse-Geisser estimates of sphericity* (Ԑ = 0.56).* Pairwise comparisons with a Bonferroni correction were calculated for post-hoc comparisons.

As a manipulation check of the effectiveness of the task difficulty, a 2 x 3 (easy view, hard view x no count, easy count, hard count) MANOVA was calculated for the dependent variable main differences found. For the number completed counting steps, a 2 x 3 (easy count, hard count x free view, easy view, hard view) MANOVA was calculated.

## Results

### Effectiveness of Task Difficulty

**Figure 3.**
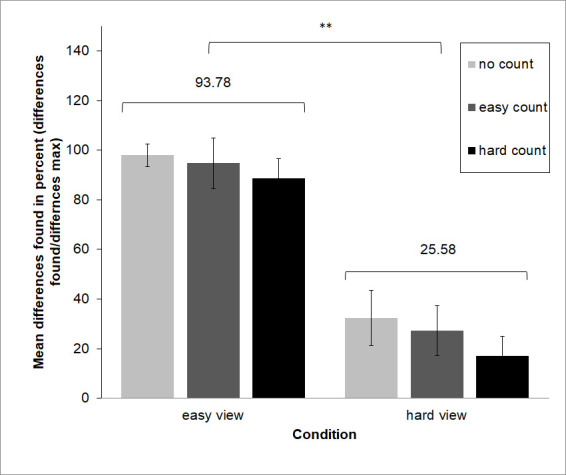
Manipulation checks for levels of difficulty suggest that both difficulty manipulations were effective. Participants found a significantly higher percentage of available changes in the easy view condition (M = 93.78%), as compared to the hard view condition (M = 25.58%), and irrespective of count condition.

**Figure 4.**
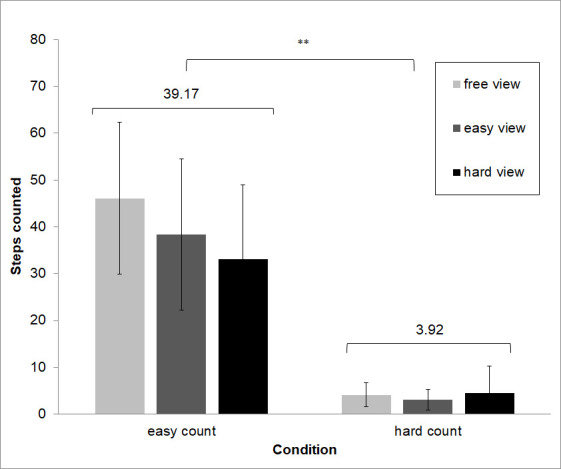
Manipulation checks for levels of difficulty suggest that both difficulty manipulations were effective. Participants likewise completed significantly more counting steps in the easy count condition (M = 39.17) than they did in the hard count condition (M = 3.92), and irrespective of view condition.

Our manipulation check indicated that the experimental manipulations were successful (see Figure 3 and 4). Participants reported a significantly higher percentage of differences in the easy condition (M = 93.78, SD ± 0.96) as compared to the hard condition (M = 25.78, SD ± 1.02), and irrespective of count condition F(1, 21) = 5040.68, *p* < .001). Participants likewise completed significantly more counting steps in the easy count condition (M = 39.17, SD ± 16.10) than they did in the hard count condition (M = 3.92, SD ± 3.5), irrespective of view condition (F(1,21) = 222.07, *p* < .001).

As a further indicator of a successful manipulation of task difficulty, subjective ratings of workload were recorded. In accord with measures of task performance, the NASA-TLX scales indicated a successful manipulation of task difficulty (see Table 1).

**Table 1:** Subjective rating of task difficulty.

	Conditions									
NASA-TLX	Free view and no count	Free view and easy count	Free view and hard count	Easy view and no count	Easy view and easy count	Easy view and hard count	Hard view and no count	Hard view and easy count	Hard view and hard count	
NASA Mental	1.15 (0.533)	5.29 (4.117)	13.68 (5.466)	4.11 (3.688)	9.21 (4.856)	14.43 (5.062)	8.02 (4.916)	12.23 (5.64)	15.97 (4.499)	
NASA Physical	1.08 (0.319)	2.11 (2.78)	3.67 (5.821)	1.70 (1.673)	2.76 (3.415)	3.69 (5.446)	2.48 (3.226)	3.55 (4.608)	4.11 (5.644)	
NASA Temporal	1.06 (0.240)	5.88 (4.728)	10.68 (6.157)	5.08 (4.193)	9.06 (5.329)	11.34 (5.840)	8.41 (5.335)	10.45 (5.977)	12.97 (6.351)	
NASA Performance	1.24 (0.878)	5.83 (3.827)	12.11 (5.203)	3.61 (2.860)	6.55 (3.216)	10.81 (4.043)	7.70 (4.102)	8.94 (3.831)	14.18 (3.847)	
NASA Effort	1.23 (0.908)	7.45 (5.745)	13.97 (5.253)	5.68 (4.651)	10.52 (5.210)	13.89 (5.466)	9.41 (5.230)	12.15 (5.148)	14.85 (5.310)	
NASA Frustration	1.15 (0.533)	5.32 (5.196)	10.11 (6.483)	3.00 (2.449)	6.33 (4.747)	9.53 (6.350)	5.79 (4.741)	8.11 (5.447)	11.32 (6.624)	

Note. Values are mean ± SD (n = 18). All scales are from the NASA-TLX (NASA-Task Load Index).

### Visual load and Microsaccade Rate

**Figure 5.**
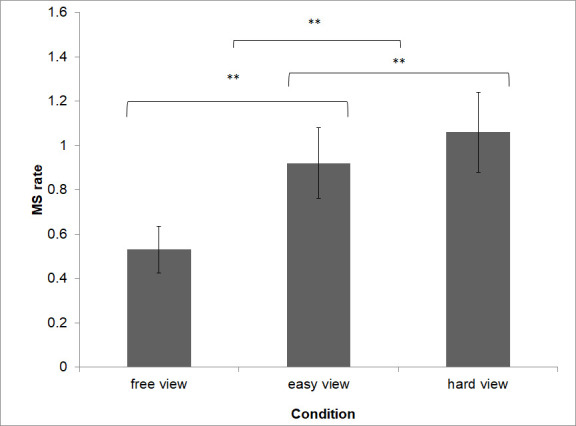
Microsaccade rate was significant higher in the hard view condition than in the easy view or free view condition.

A significant main effect of visual load on microsaccade rate (F(1.12, 23.68) = 24.62, *p < *.001) was evident (Figure 5). The pairwise comparisons (corrected using Bonferroni adjustments) indicate that the significant main effect reflects a significant difference (*p *< .001) between condition ‘free view‘ (*M *= 0.53 SD ± 0.10) and ‘easy view‘ (*M *= 0.92, SD ± 0.15) and ‘easy view‘ (*M *= 0.92, SD ± 0.15) and ‘hard view‘ *(M = *1.09, SD ± 0.18) and ‘hard view‘ *(M = *1.09, SD ± 0.18) and ‘free view‘ (*M *= 0.53 SD ± 0.10). Microsaccade rate increased with increasing task difficulty of the visual task (linear trend: F(1, 21) = 28.19, *p* < .001, see Figure 5).

**Figure 6.**
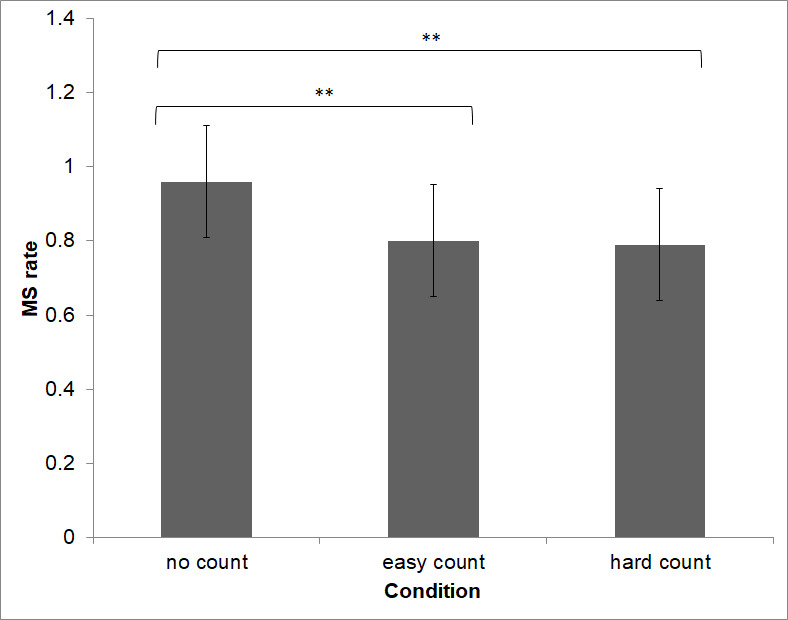
Microsaccade rate decreased as task difficulty increased in the mental workload task. In the no count condition microsaccade rate was significantly higher than in the easy count or hard count condition.

With regard to the manipulation of mental demand, results indicated a significant main effect on microsaccade rate (F(1.49, 31.48) = 5.80, *p < .*01, see Figure 6). Post-hoc comparisons indicated that microsaccade rate changed significantly between the ‘no count‘ (*M *= 0.96, SD ± 0.14) and the ‘easy count‘ (*M* = 0.80, SD ± 0.14) condition (*p* < .01) and the ‘no count‘ and ‘hard count‘ (*M *= 0.79, SD ± 0.15) condition (*p *= .02). However, no significant change in microsaccade rate was found between the ‘easy count‘ and ‘hard count‘ condition (*p *= .82).

The interaction between visual demand and mental demand was not significant (F(2.85, 60.04) = 2.64, *p*= .06).

**Figure 7.**
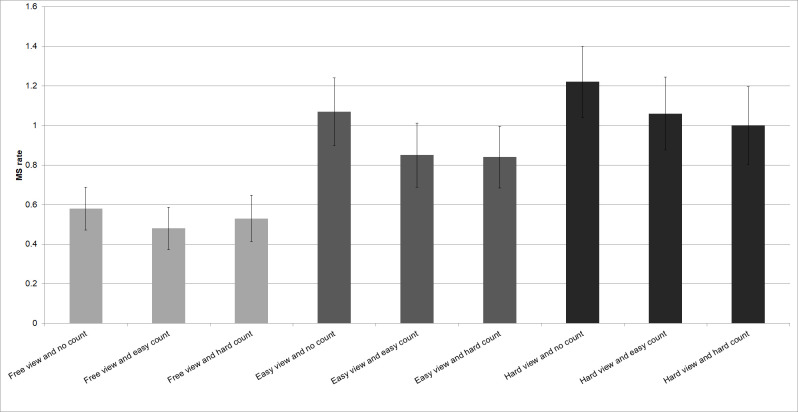
Microsaccade rate decreases when attention is directed towards the mental load task. Opposite, microsaccade rate increases when attention is directed towards the visual load task.

Figure 7 shows that Microsaccade rate increased in high visual load conditions. Microsaccade rate decreased in conditions that required high mental demand when attention was directed towards the cognitive load task. Microsaccade rate increased when attention was directed towards the visual load task.

## Discussion

Our results show that microsaccade rate reflects the amount of visual attention toward a visual task. For demanding tasks, this suggests the utility of microsaccade rate as a biomarker of whether an operator is just gazing an object or if they have really focused their attention. In this, our hypothesis was upheld, as trials with increased visual load (‘easy‘ or ‘hard view‘ task) did result in increased microsaccadic rates, relative to trials with low visual load (‘free view‘ task). Trials with high demand visual tasks also increased microsaccadic rates more than those with low demand visual tasks. These results are in accordance with 15 and 16. Also, our hypothesis was upheld, since tasks inducing cognitive load (‘easy count‘ or ‘hard count‘) alone would result in decreased microsaccadic rates. Likewise, trials with high demand cognitive tasks decreased microsaccadic rates more than those with no demand cognitive tasks. These findings are in accordance with 17, 18 and 19. However, contrary to 17 we didn’t find a linear effect but only a general load effect. There was no significant effect between easy count and hard count. Beyond replicating past results, the present data show that microsaccade rate rather granularly reflects the difficulty of visual stimuli. Indeed, it may in fact reflect how much attention is directed to a visual task, and how much of the visual information is processed. As such, microsaccades may well be useful in applied settings to indicate how much attentional capacity is directed toward a visual target, if indeed any.

### Measuring Visual Load

The present results show that the visual demand of a task is systematically reflected in microsaccade rate (Figure 5). Any single visual task (‘easy‘/‘free‘/‘hard view‘ task combined with ‘no count‘ task) showed an increased microsaccade rate compared to its comparator in a dual task setting (Figure 7). Also, all ‘hard view‘ condition tasks show an increased microsaccade rate compared to all ‘easy view‘ condition tasks. The explanation of these results is that in a single visual task the operator shifts his full attention to that visual task. A ‘hard view‘ condition task, inducing more visual load, requires more visual attention reflected by a higher microsaccade rate. However, when the visual task is combined with a mental task (dual task setting), the microsaccade rate decreases significantly. The underlying explanation here is that the second non visual task requires a certain amount of attention. In consequence, the operator does not direct his full working memory capacity which is shifted towards the visual task.

### Limitations

The difference in microsaccade rate between the ‘easy count‘ and ‘hard count‘ task was not significant. It seems likely that in this case there was a floor effect, since the hard count task was indeed ‘hard‘ for the participants. Indeed, anecdotally, participants found our task of counting backwards by 17s so difficult that they sometimes just gave up. Another possible explanation is that pushing the button in our MCT task required resources relevant to our DVs of interest, and so had some systematic influence. In the ‘easy count‘ condition participants pushed the button more often than in the ‘hard count‘ condition. Also, it is important to remember that the aggregate difficulty of difficult visual and cognitive demand may not be additive, but multiplicative. Other studies with a constant visual task showed a similar effect to this study (17).

Of course, more work is needed to understand both the import and full meaning of the present pattern of data. Very little, one must remember, is known about microsaccadic activity, especially in rich visual stimuli like that used in the present effort. Indeed, higher microsaccade rates shown in the present study might simply be the result of some artifact of our stimuli set; for example, fine detail on the picture. The higher rate of occurrence of microsaccades in the hard view condition could be due to task-related demands, but also because there are more small features in the ‘hard view‘ condition task. The effect could be partially bottom-up and not only determined by the difficulty of the change detection task.

### The distribution of attentional processes

According to the present results microsaccade rate is modulated by the visual information processing (and visual attention) and a certain microsaccade level is required for minimal visual attention. As a consequence, the decrease in the microsaccade rate demonstrates a limited capacity for simultaneous attentional processes in different modalities (i.e. visual vs. non-visual). In everyday life humans deal with visual information simultaneously while dealing with other non-visual information (i.e. mental processes, acoustic-, tactile-, or olfactory- information). A very common example would be in driving a car and simultaneously making a phone call. The decision as to what information is processed is reflected in the distribution of that attention. Working memory has a central role in this distributional process and absolute and relative microsaccade rate could help to specify these attentional shifts (19). Further, they could give insight into the neurological conceptions of working memory and the distribution of attentional processes.

### Importance in Practical Settings

A measure that monitors visual attention and to what extent an individual is processing the associated visual information is of critical importance. Not only will basic research benefit from this knowledge, but also vast swathes of applied investigation will profit since inattention to visual cues frequently lead to errors and accidents. The example given in the introduction; a car driver who doesn’t register a signal turning green, might appear to be a rather benign example. But consider a car driver not registering a green signal turning to red. Or consider an educational setting. A teacher may draw student pupils` attention to a certain visual location, but if the student simply ‘looked but did not see‘ then the next steps in the learning sequence may be negated as the thread of learning lost; all the while the teacher might feel assured that they had sufficiently featured the item so that they assumed fixation had equated with content processing. In such cases, inattention directly leads to failure.

Having a measure for visual attention and visual information processing might distinguish between ‘looking‘ and actually ‘seeing‘. Especially where safety is a function of attention (i.e. traffic safety, aviation safety, patient safety etc.) the significance and benefits of such a measure should be clearly evident. Indeed, such a measure could provide real-time feedback as to how much an individual is spending their attention on a visual task. For example, it could provide feedback on how much a car driver is visually focused on the street and relevant surrounding and signals and it would give feedback whenever the attention is shifting to non-driving displays (i.e. to mental processes) (35). At the moment there exists no unequivocal physiological measure for visual attention or visual information processing. Indeed, even at a time when the visual fixation of an object has been shown unequivocally to not necessarily be equated with focusing attention toward that object, there are still systems which use this logic, presumably for lack of something better. For example, Chevrolet’s SuperCruise, a production self-driving technology, uses measures of gaze to the roadway to enforce eyes-on-road during autonomous driving. How much better to enforce attention-to-driving-task, given the technological means!

Although there has been extensive and prolonged use of certain visual processing measures, the specifics of the idea to include fixational eye movements (i.e. microsaccades) is a relatively new one. Microsaccades are typically investigated in neurological settings and are interesting measures since they are mostly not consciously controlled. One procedural problem is the infrastructure needed for detecting microsaccades. High-speed eye tracking devices are typically non-mobile and not suitable for applied settings beyond evaluation in simulators. Since there is obviously empirical evidence that microsaccades are an adequate measure for visual information processing, the development of mobile high-speed eye tracking systems will hopefully progress. This would open a new field in many real-world settings.

## Conclusion

In the same way that vagal tone has been represented as being responsive to variations in cognitive load (36), we have proposed and confirmed here that inhibition in microsaccade rate accompanies increases in cognitive demand. As with the vagal connection, we also recognize that microsaccades, most probably, do not subserve a one single function. However, it is evident that such measures do provide a window into cognitive state and that clarity of that window (i.e., the signal to noise ratio of this specific measure) is high. This makes microsaccade rate observation an exceptionally useful and diagnostic tool in the evaluation and prediction of real-world behavior.

Our results indicate that the microsaccade rate can reflect both the level of visual attention and the level of visual information processing. A measure that monitors how and to what extent an individual is focused on a specific visual task is this a critical step for the application of visual assessment to real world tasks. More research is necessary to see whether the paradigm works in a variety of ever more applied field settings and the degree to which the resultant signed can be fed-back into cybernetic control systems for human-machine interface and exchange. More work is needed on the basic vision-science, where significant gaps in our understanding of microsaccades remain. The reward will be a measure which reflects to what extent and how an operator is processing visual information, a critical step for both experimental work to understand multitasking, and toward the application of sophisticated visual assessment to real world tasks.

## Ethics and Conflict of Interest

The author(s) declare(s) that the contents of the article are in agreement with the ethics described in http://biblio.unibe.ch/portale/elibrary/BOP/jemr/ethics.html and that there is no conflict of interest regarding the publication of this paper.
